# Thermodynamic Origin of Negative Thermal Expansion Based on a Phase Transition-Type Mechanism in the GdF_3_-TbF_3_ System

**DOI:** 10.3390/ijms241914944

**Published:** 2023-10-06

**Authors:** Elena A. Sulyanova, Boris P. Sobolev

**Affiliations:** Shubnikov Institute of Crystallography, Federal Scientific Research Centre “Crystallography and Photonics”, Russian Academy of Sciences, Leninskiy Prospekt 59, 119333 Moscow, Russia

**Keywords:** negative thermal expansion, polymorphic transformation, rare-earth trifluorides, phase diagrams, phase rule, principle of continuity

## Abstract

Multicomponent fluorides of *rare earth elements* (**REE**s—***R***) are *phase transition-type negative thermal expansion* (**NTE-II**) materials. NTE-II occurs in *R*F_3_-*R′*F_3_ systems formed by “mother” single-component dimorphic *R*F_3_ (*R* = Pm, Sm, Eu, and Gd) with a giant NTE-II. There are two structural types of *R*F_3_ polymorphic modifications: low-temperature β-YF_3_ (***β***−) and high-temperature LaF_3_ (***t***−). The change in a structural type is accompanied by a density anomaly: a *volume of one formula unit* (**V_form_**) V***_β_***_−_ >V***_t_***_−_. The empirical signs of volumetric changes ΔV/V of NTE-II materials were considered. For the GdF_3_-TbF_3_ model system, an “operating-temperature window Δ***T***” and a two-phase composition of NTE-II materials follows from the thermodynamics of chemical systems: the phase rule and the principle of continuity. A necessary and sufficient sign of NTE-II is a combination of polymorphism and the density anomaly. Isomorphism in *R*F_3_-*R′*F_3_ systems modifies *R*F_3_ chemically by forming two-component ***t***− and ***β***− type *R*_1−x_*R’*_x_F_3_ *solid solutions* (***ss***). Between the two monovariant curves of *ss* decay, a two-phase area with Δ***T***_trans_ > 0 (the “window Δ***T***”) forms. A two-phase composite (***t***−*ss* + ***β***−*ss*) is an NTE-II material. Its constituent ***t***−*ss* and ***β***−*ss* phases have different V_form_ corresponding to the selected ***T***. According to the lever rule on a conode, V_form_ is calculated from the ***t***−*ss* and ***β***−*ss* compositions, which vary with ***T*** along two monovariant curves of *ss* decay. For the GdF_3_-TbF_3_ system, ΔV/V = ***f***(***T***), ΔV/V = ***f***(Δ***T***) and the “window Δ***T***” = ***f***(*x*) dependencies were calculated.

## 1. Introduction

The search for new materials with *negative thermal expansion* (**NTE**) has been conducted via trial and error for many years. Recently, there has been a noticeable change in the paradigm for creating NTE materials. Chemical modification of known “mother” materials with a giant NTE was used [1]. The method of chemically modifying “mother” substances was called “substitutions” [1]. This implies isomorphism, which can be realized in a binary (and more complex) system.

The development and application of multicomponent fluoride materials with the participation of REE trifluorides—*R*F_3_—have been carried out since the mid-1970s [2,3,4]. No work has been performed on the search for and study of NTE materials based on REE fluorides.

The only described REE trifluoride with NTE is ScF_3_ [5,6,7,8]. It belongs to the 1st type, **NTE-I**, which includes conventional materials with monotonous volume reduction when heated over a wide temperature (***T***) range [1]. Owing to its simple composition and cubic structure, a special mechanism of NTE-I was discovered for ScF_3_ [5,6,7,8]. ScF_3_-*R*F_3_ systems have not been extensively studied.

The 2nd type of NTE (**NTE-II**) includes materials with NTE based on a phase transition-type mechanism. In such materials, a volume reduction when heating occurs during a *polymorphic transformation* (**PolTr**) [1].

*R*F_3_-*R*′F_3_ systems are the most suitable for studies of the common signs of NTE-II materials. They are ionic compounds with the simplest formula and high chemical stability. Their melting (***T***_fus_) and PolTr (***T***_trans_) temperatures ensure diffusion processes and the achievement of equilibrium.

REE trifluorides form a long homologous series of *R*F_3_ (*R* = La − Lu) from extremely chemically similar compounds with a minimum difference in the *atomic numbers* (**Z**) of cations in the series (Δ*Z* = 1). The lanthanide compression of cations in the *Ln*F_3_ (*Ln* = Ce − Lu) series leads to three types of structures and two *morphotropic transformations* (**MorphTrans**).

*R*F_3_ are polymorphic [9]. They (with the exception of volatile ScF_3_) crystallize in three structural types: LaF_3_ (***t***−) [10,11], β-YF_3_ (***β***−) [12], and α− UO_3_ (***α***−) [13]. There are three possible PolTrs in *R*F_3_-*R′*F_3_ systems: ***β***− → ***t***−, ***β***− → ***α***−, and ***t***− → ***α***−.

Among 17 *R*F_3_s, four dimorphic fluorides have been discovered: PmF_3_, SmF_3_, EuF_3_, and GdF_3_ [2]. SmF_3_, EuF_3_, and GdF_3_ were described as having a density anomaly: a high-temperature form is denser than a low-temperature form [14]. Recently, PmF_3_ was attached to them [15]. The density anomaly is the main structural feature of PolTr in dimorphic *R*F_3_s. This makes them and *R*F_3_-*R*′F_3_ systems, which are formed by them, sources of NTE-II materials.

In this message, a new paradigm for the design of NTE-II materials using isovalent isomorphism [1] is considered based on phase diagrams.

In [16], the principle of isovalent isomorphism in *R*F_3_-*R*′F_3_ systems with one (or two) components with NTE-II was used to predict two-component two-phase compositions with NTE-II in 50 (out of 105) systems. Four stages for creating two-component NTE-II materials with adjustable operational parameters were considered. For systems with the studied phase diagrams, the temperature and composition ranges in which these parameters are regulated were estimated.

The connection of empirically found features of two-component NTE-II materials with the thermodynamic rules for the design of phase diagrams of chemical systems, Gibbs’ phase rule (1879) and Kurnakov’s continuity principle [17] (1936), is established.

It is shown that NTE-II follows from the fundamental laws of thermodynamics of chemical systems. In this study, the empirical signs of NTE-II [1] in a material based on its phase diagram are substantiated for the first time on the model GdF_3_-TbF_3_ system [2]. One of its components, GdF_3_, has a giant NTE-II.

A comparison of the rules for the phase diagram design with the thermophysical properties—*coefficients of thermal expansion* (**CTE**) of the materials formed in them—defines the universal signs of NTE-II.

The compliance of the formation of NTE-II materials with the fundamental rules of chemical thermodynamics provided a reasonable correction of their previously found empirical features.

A phase diagram of a system contains all the data for quantitative calculations of the values of the NTE-II parameters of the materials formed in this system. This report presents a scheme for calculating the parameters of an NTE-II material based on the experimental phase diagram of the selected GdF_3_-TbF_3_ system.

This scheme can be extended to any system with a studied phase diagram if it is equilibrium and if its components (one or both) are compounds with NTE-II.

*The aims of this study* are as follows: (1) a discussion of NTE-II empirical signs in one- and two-component fluoride materials on the basis of the fundamental rules for the chemical system design (the phase rule and the principle of continuity); (2) formulating the stages of the design of new fluoride NTE-II materials with adjustable parameters with isomorphic substitutions in “mother” *R*F_3_ (*R* = Pm, Sm, Eu, and Gd) with PolTr (when heated); (3) consideration of the thermodynamic conditions (an “operating-temperature window Δ***T***” and a two-phase composite) for the NTE-II material formation in the model GdF_3_-TbF_3_ system; (4) and a presentation of the method for calculating the NTE-II parameters controlled by isomorphism from the phase diagram of the GdF_3_-TbF_3_ model system.

According to IUPAC, 17 REEs were designated *R*: Sc, Y, La and 14 *Ln* = Ce − Lu. When analyzing the periodicity of the properties of *Ln* compounds, it is necessary to separate La from the 4*f* elements *Ln*. If not necessary, the sum (La + *Ln*) was denoted by *R*. The inconvenience of the REE classification was noted by the IUPAC project (2015) regarding the La position.

Subscript designations of compositions in chemical formulas make it advisable to assign *Z* of *R* in the superscript position (^57^Ce_0.5_^64^Gd_0.5_F_3_). Atomic weights with generally accepted superscript positions are not used in this work.

## 2. Results and Discussion

### 2.1. Signs of NTE-II Materials

#### 2.1.1. The Qualitative Description of the NTE-II Materials Signs

In the literature, one can find the opinion that a NTE-II material must (1) be two-phase, (2) have the “window Δ***T***”, and (3) have the negative CTE that can be measured within the “window Δ***T***”.

The thermal expansion law has the following form:

ΔV/V = α_V_ Δ*T*. (1)

The proportionality coefficient in the linear dependence (1) represents the volumetric CTE α_V_ = dV/d***T***. It characterizes the rate of change, ΔV/V, as a function of ***T***. NTE-II materials have negative α_V_ within the “window Δ***T***”, which can be up to 10 times greater than that of conventional materials [1]. The total volume change **ΔV/V** was used in the literature to compare the densities of compounds with different structures. It is recommended [1] as an intrinsic index to indicate the potential of NTE-II.

**Figure 1:**
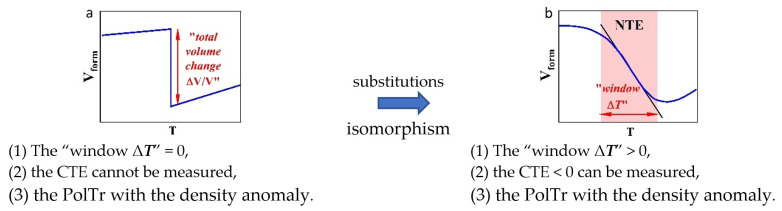
Comparison of the NTE-II schemes for (**a**) one-component and (**b**) two-component (substitutions” = isomorphism) NTE-II materials.

Qualitative schemes of the ΔV/V change from ***T*** for an NTE-II material according to [1] are presented in Figure 1. The diagram in Figure 1a demonstrates the PolTr occurring “at a point” (***T***_trans_ = const). At ***T***_trans_, ΔV/V jumps at Δ***T*** = 0 (the “window Δ***T***” = 0) [1]. The scheme in Figure 1a from [1] corresponds to the phase rule for a single-component condensed system. The “window Δ***T***” is prohibited by the phase rule.

During PolTr in a single-component system, CTE cannot be measured, because Δ***T*** at the point of the PolTr is zero. The author [1] draws a fundamental conclusion from this: “The coefficients α_V_ and α_L_ are not intrinsic for NTE-II materials. The ΔV/V is the intrinsic index of the NTE potential”.

The diagram in Figure 1a corresponds to simple “mother” substances, showing PolTr with the density anomaly of the modifications [1].

When the second component is added to a “mother” [1] simple substance, an isomorphic substitution of some of the cations occurs with the formation of *ss*. The term “substitutions” (Figure 4 in [1]) indicates isomorphism. The degree of freedom of a system increases by 1. The “window Δ***T***” > 0 appears, and the vertical line in the diagram (Figure 1a) acquires a *slope* (Figure 1b). In a binary system, PolTr occurs over a temperature range. If within the “window Δ***T***” a material has CTE < 0 that can be measured, such a material belongs to materials with NTE-II.

For a single-component dimorphic compound, ΔV/V, at the PolTr, is an intrinsic constant of a substance. The concept of the “total volume change” is applicable to it as a characteristic of PolTr.

The ΔV/V of a multicomponent material in a binary (and more complex) system generally varies with composition. Exceptions are the singular points of a phase diagram with invariant equilibria.

The “window Δ***T***” between two phases exists in any binary system with *ss* on the base of both structural modifications of components. This corresponds to the phase rule, not to the proof of the presence of NTE-II in a material. These are signs of a condensed ***T–x*** system with *ss*, based on its components. Many substances are polymorphic. Polymorphism is also not a sign of NTE-II.

In Figure 1, PolTr with the density anomaly is shown. The PolTr of a one-component material (Figure 1a) proceeds with the density anomaly and the “window Δ***T***” = 0. The PolTr of a two-component material (Figure 1b) also proceeds with the density anomaly but with the “window Δ***T***” > 0.

The qualitative signs of NTE-II, formulated based on empirical data, need to be tested on experimental systems with such materials. This is what the present study is dedicated to.

A necessary and sufficient condition for NTE-II is polymorphism combined with the density anomaly. Changing the structure with a decrease in ΔV/V at the PolTr (when heated) is of key importance for the emergence of NTE-II.

#### 2.1.2. Signs of NTE-II in Ionic REE Fluorides

All *R*F_3_, except for volatile ScF_3_, are single-component condensed systems. Their PolTrs are invariant processes with ***T***_trans_ = *const* and the “window Δ***T***” = 0, which are described by the scheme in Figure 1a.

The proposed [1] chemical modification of “mother” simple *R*F_3_ with the PolTr and NTE-II by isomorphism can be obtained only in a binary (and more complex) system, forming two-component NTE-II materials. Such materials are formed in *R*F_3_-*R′*F_3_ systems.

The objectives of this study include the construction of the qualitative and quantitative schemes of the PolTr in these systems using the example of the GdF_3_-TbF_3_ system. The bank of phase diagrams of 34 studied *R*F_3_-*R′*F_3_ systems [2] serves as a scientific basis for this message.

Let us consider the signs of NTE-II materials in *R*F_3_-*R′*F_3_ systems based on empirical data from the literature.

Polymorphism is the first unconditional sign of any NTE-II material [1]. The connection with PolTr defines the type of NTE material as the 2nd type [1]. PolTr separates NTE-II from a conventional NTE (compression when heated over a wide ***T*** interval) and from normal materials (expansion when heated).

When investigating polymorphism, it is necessary to consider the peculiarities of this phenomenon. These are reflected in the discussion of polymorphism in IUPAC. It was said that its absence was most often caused by “a lack of financial resources for research”. These are state parameter changes in the areas of high pressures and low temperatures, which incur large financial costs. Under such conditions, polymorphism can remain “hidden”.

PmF_3_ polymorphism is a vivid confirmation. Microquantities of PmF_3_ were obtained in a nuclear reactor according to the Manhattan Project to determine its structural affiliation with the LaF_3_ type [18]. The cost of the reagent was not determined. However, its high cost has contributed to the lack of knowledge concerning PmF_3_.

PmF_3_ polymorphism still remains “hidden” in terms of thermal analysis techniques. This occurs at low temperatures and is accompanied by a small thermal effect [15]. Only the method of structural and chemical modeling allowed the evaluation of ***T***_trans_ in the model ^61^(Ce_0.5_Gd_0.5_)F_3_ (“pseudo ^61^PmF_3_”) composition [15].

Polymorphism is a necessary but insufficient feature of NTE-II. Of the eight dimorphic *R*F_3_, only four (*R* = Pm, Sm, Eu, and Gd) possess a giant ΔV/V.

The second sign of NTE-II is the density anomaly at the PolTr. Under comparable conditions, the **V_form_** (the volume of one formula unit) of a high-temperature modification is lower than that of a low-temperature modification, V_low_ (V_high_ < V_low_, compression when heated).

Only ***β***− → ***t***− PolTr (when heated) of the three possible structural transitions yields a denser high-temperature ***t***−modification. The volumes of dimorphic *R*F_3_ are V***_β_***_−_ > V***_t_***_−_ (V***_t_***_−_ and V***_β_***_−_ are V_form_s of ***t***− and ***β***− types, respectively). In single-component SmF_3_, EuF_3_, and GdF_3_, density anomalies were noted for the first time [14].

Polymorphism and the density anomaly are determined by structural changes that occur during PolTr. Structural changes are key to understanding the NTE-II mechanism. In [1], the density anomaly as a sign of NTE-II was not discussed. This is the main structural limitation of NTE-II.

The third sign of NTE-II is a two-phase composition [1]. In a single-component *R*F_3_ system, a two-phase area is prohibited by the phase rule. A two-phase area forms in binary systems between the two areas of homogeneity of the ***β***-*R*_1−x_*R′*_x_F_3_- *ss* and ***t***-*R*_1−x_*R′*_x_F_3_-*ss* based on *R*F_3_ modifications.

The fourth sign of NTE-II is the “*window* Δ***T***”, according to [1]. The origin of this feature is not established [1]. The “window Δ***T***” defines the second fundamental parameter of an NTE-II material. Its significance in the description and use of NTE-II materials plays a dual role. First, the “window Δ***T***” controls ΔV/V in a particular system, which determines the use of a material. Control of ΔV/V is considered in the example of the GdF_3_-TbF_3_ system in this study. Secondly, the “window Δ***T***” “adjusts” a material to the temperature conditions of use. For one system, the value of the regulated Δ***T*** is small. In *R*F_3_-*R′*F_3_ systems, the position of the “window Δ***T***” on the ***T*** scale can vary widely from low temperatures to melting.

The fifth sign of NTE-II is a measurable CTE. It is present only in two-component materials formed in *R*F_3_-*R′*F_3_ systems because their PolTrs occur over a temperature range Δ***T***. In a single-component *R*F_3_, CTE cannot be measured at PolTr (Δ***T*** = 0).

To realize NTE-II in dimorphic ionic fluorides *R*F_3_ with *R* = Pm-Gd, their structural types must have features. The nature of the structural mechanism of NTE-II in *R*F_3_ has not yet been clarified.

### 2.2. Two-Component NTE-II Materials with Adjustable Parameters in the GdF_3_-TbF_3_ System

**Figure 2:**
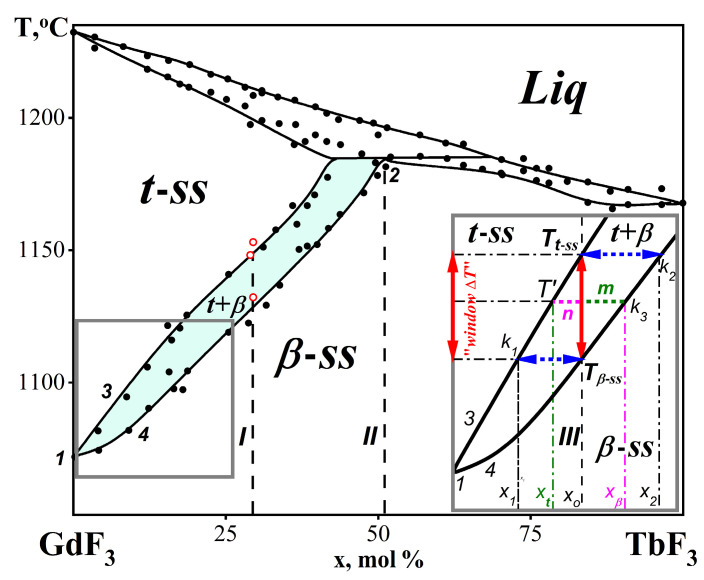
The GdF_3_-TbF_3_ phase diagram: (1) the PolTr of GdF_3_ with Δ***T***_trans_ = 0, (2) the incongruent melting ***β***-Gd_0.49_Tb_0.51_F_3_, (3) the ***t***−*ss* decay curve, and (4) the ***β***−*ss* decay curve. Insert: The scheme for the calculation of the qualitative and quantitative composition of the *two-phase* (***β*** + ***t***)-Gd_1−x_Tb_x_F_3_ composite. The experimental data are shown as closed black and open red (x ≈ 0.29) circles, “window Δ***T***” intervals as vertical red arrowed lines, concentration intervals as horizontal blue arrowed lines, and the two-phase (***β*** + ***t***)-Gd_1−x_Tb_x_F_3_ area in light green.

The GdF_3_-TbF_3_ system was selected as a model [2] to describe the thermodynamic mechanism of the formation of NTE-II materials. Its phase diagram is shown in Figure 2. The black points represent thermal effects determined experimentally using the thermal analysis method.

#### 2.2.1. Choosing a Model System

The first component of the GdF_3_-TbF_3_ model system, dimorphic GdF_3_, has a giant NTE-II (ΔV/V ~ 4.3%) [14,19] at the ***β***-GdF_3_ → ***t***-GdF_3_ PolTr (when heated). The choice of GdF_3_ from *R*F_3_ (*R =* Pm, Sm, Eu, and Gd) is due to its stability in terms of valency reduction.

The chemical modification of GdF_3_ due to isomorphism occurs when TbF_3_ is added. A new state parameter*,* composition *x* (mole % of TbF_3_), appears in the GdF_3_-TbF_3_ system. This increases the degree of freedom of the system by 1.

The PolTrs in two-component systems are a combination of peritectoid (one phase turns into two phases with an increase in ***T***) and eutectoid (two phases turn into one phase with an increase in ***T***) equilibria. This is accompanied by the formation of a two-phase area [20].

According to the principle of continuity [17], two-component ***β***-Gd_1−x_Tb_x_F_3_ and ***t***-Gd_1−x_Tb_x_F_3_ phases form the two-phase area (***β*** + ***t***)-Gd_1−x_Tb_x_F_3_ with the *ss*, which have the ***β***− and ***t***− structural modifications of one-component GdF_3_. The properties of the ***β***− and ***t***− modifications change continuously when moving from the single- to two-phase area.

The GdF_3_-TbF_3_ system has a convenient interval of thermal effects for the thermal analysis method, as set by the difference Δ(***T***_fus_-***T***_trans_) of GdF_3_. This made it possible to study phase diagrams that contain a high frequency of the analyzed compositions (the black dots and red circles in Figure 2) when obtaining both liquidus and solidus curves.

Curves 3 and 4 of the Gd_1−x_Tb_x_F_3_ *ss* decay (formation) in Figure 2 come from point 1 (the PolTr of GdF_3_). The Gd_1−x_Tb_x_F_3_ *ss* have the same types of structures (***β***− and ***t***−) that lead to the giant NTE-II in GdF_3_. Anomalous volume relations remain between them: V***_β_***_−*ss*_ > V***_t_***_−*ss*_ (the compression occurs with an increase in ***T***).

The homogeneity area of the two-phase composite (***β*** + ***t***)-Gd_1−x_Tb_x_F_3_ with NTE-II captures approximately 50 mol% of the TbF_3_ composition axis. It is marked in Figure 2 in light green.

The Δ(***T***_fus_ − ***T***_trans_) = 1228–1070 °C interval (the GdF_3_ phase transformations) from the phase diagram [2] lies in the ***T*** region of disinhibited volumetric diffusion in a solid (Tamman’s temperature is ***T***_tamm_ ~ 760 °C). This ensured an equilibrium necessary for the use of materials with NTE-II.

MorphTrans (Figure 2) occurs in the GdF_3_-TbF_3_ system [2]. This MorphTrans is the first in the *R*F_3_ series. It is a true MorphTrans (according to V.M. Goldschmidt, 1925) and is realized via an invariant peritectic reaction:

***Liq*** + ***t***-Gd_0.57_Tb_0.43_F_3_ ↔ ***β***-Gd_0.49_Tb_0.51_F_3_

(point 2 in Figure 2) at ***T***_trans_ = *const* = 1186 ± 10 °C [2]. The dotted vertical line *II* in Figure 2 corresponds to the point of nonvariant composition.

#### 2.2.2. The Method of Calculating the Phase Composition of an NTE-II Material on the Example of the GdF_3_-TbF_3_ Phase Diagram

The determination of the qualitative and quantitative compositions of a two-phase (***β*** + ***t***)-Gd_1−x_Tb_x_F_3_ composite is shown in the insert of Figure 2. It is defined by the isothermal sections (conodes) *k*_1_, *k*_2_, and *k*_3_. The ***β***-Gd_1−*x*o_Tb*_x_*_o_F_3_ → ***t***-Gd_1−*x*o_Tb*_x_*_o_F_3_ PolTr (when heated) of the Gd_1−*x*o_Tb*_x_*_o_F_3_ *ss* with the composition *x*_o_ begins at ***T_β_***_−***ss***_ (the *k*_1_ conode) and ends at ***T_t_***_−*ss*_ (the *k*_2_ conode).

##### The Single-Phase ***β***-Gd_1−x_Tb_x_F_3_-*ss* Area without NTE

The figurative point, which corresponds to the gross composition, *x*_o_, moves up (with the increase in ***T***) along the vertical line, *III*, in the single-phase ***β***−*ss* area to curve 4. In this area, a material is single-phase. It has a ***β***− type structure and is a conventional material without NTE.

##### The Two-Phase (***β*** + ***t***)-Gd_1−x_Tb_x_F_3_ Area with NTE-II

At the temperature ***T_β_***_−***ss***_, the figurative point intersects with curve 4. The decay of the ***β***-Gd_1−x_Tb_x_F_3_ phase begins with the release of the ***t***-Gd_1−x_Tb_x_F_3_ phase. The two-phase composite (***β*** + ***t***)-Gd_1−x_Tb_x_F_3_ is formed. The area of the two-phase composite is limited by curves 3 and 4. This is highlighted in light green in Figure 2.

The composition (*x*_1_) of the ***t***-phase at the beginning of the decay is determined by the intersection of the *k*_1_ conode (the lower horizontal dotted line) with curve 3 (insert in Figure 2).

When moving the figurative point inside the two-phase area along the vertical red arrowed line from ***T_β_***_−*ss*_ to ***T_t_***_−*ss*_ (insert in Figure 2), the composition of ***t***-Gd_1−x_Tb_x_F_3_ varies from *x*_1_ to *x*_o_. The composition of ***β***-Gd_1−x_Tb_x_F_3_ varies from *x*_o_ to *x*_2_.

At ***T_t_***_−*ss*_ (the left edge of the *k*_2_ conode), the figurative point intersects curve 3, and the composition of ***t***-Gd_1−x_Tb_x_F_3_ becomes equal to the original one (*x*_o_). PolTr, formally similar to the ***β***-GdF_3_ → ***t***-GdF_3_ PolTr, is completed.

However, because ***β***-Gd_1−x_Tb_x_F_3_ and ***t***-Gd_1−x_Tb_x_F_3_ are located on the different monovariant curves 3 and 4 and are separated by the interval Δ***T***, the PolTr proceeds over the temperature range. The “operating-temperature window Δ***T***“ is formed [1]. In the insert in Figure 2, “window Δ***T***“ is indicated for composition *x*_o_ by the red arrow. Its value is Δ***T*** = ***T_t_***_−*ss*_ − ***T_β_***_−*ss*_.

An example of calculating the average V_form_ inside the “window Δ***T***” for the intermediate point of the PolTr at ***T***′ (conode *k*_3_) within the Δ***T*** interval is shown in the insert in Figure 2. The mole fractions (*m* and *n*) of the ***t***−*ss* and ***β***−*ss* in the two-phase (***β*** + ***t***)-Gd_1−x_Tb_x_F_3_ area are shown in green and magenta, respectively. These values are calculated according to the lever rule. The concentrations of *x**_t_*** and *x**_β_*** of the ***t***−*ss* and ***β***−*ss* correspond to the extreme points of the *k*_3_ conode.

The concentration *x*_o_ of Gd_1−x_Tb_x_F_3_ at the beginning of the PolTr at ***T_β_***_−*ss*_ corresponds to V_o_. It is equal to the V_form_ of the ***β***−*ss* (V***_β_***_−_) phase, which is 100%.

V_o_ = V***_β_***_−_(*x*_o_), (2)

V_form_ of the ***t***−*ss* (V***_t_***_−_) and ***β***−*ss* (V***_β_***_−_) phases entering the two-phase composite (***β*** + ***t***)-Gd_1−x_Tb_x_F_3_ at ***T***′ are calculated by (3) and (4):

V***_t_***_−_ = *m*V***_t_***_−_(*x**_t_***), (3)

V***_β_***_−_ = *n*V***_β_***_−_(*x**_β_***), (4)

V_form_s V***_t_***_−_(*x_t_*) and V***_β_***_−_(*x_β_*) are calculated from the unit cell parameters of the ***t***−*ss* and ***β***−*ss* phases. The unit cell parameters of the ***t***−*ss* and ***β***−*ss* phases, in turn, are determined using Vegard’s rule from the unit cell parameters of the GdF_3_ and TbF_3_ components [14,19]. The unit cell parameter of TbF_3_ was determined via the extrapolation of the *R*F_3_ data [19].

Let us assume that the NTE-II composite material represents an equilibrium mechanical mixture of its constituent ***t***−*ss* and ***β***−ss phases. Then, the V_form_ of the two-phase compositions (V***_t_*_+*β*_**) is calculated using (5) as the sum of the volumes (V***_t_***_−_ + V***_β_***_−_) of the ***t***−*ss* and ***β***−*ss* phases:

V***_t_*_+*β*_** = V***_t_***_−_ + V***_β_***_−_ (5)

The average value of V_form_ of a composite is variable. It is determined by ***T*** in the interval Δ***T***.

##### The Single-Phase ***t***-Gd_1−x_Tb_x_F_3_-*ss* Area without NTE

At ***T_t_***_−***ss***_, the figurative point intersects with curve 3. The decay of the ***β***-Gd_1−x_Tb_x_F_3_ phase with the release of the ***t***-Gd_1−x_Tb_x_F_3_ phase is completed. The figurative point falls into the ***t***-Gd_1−x_Tb_x_F_3_ single-phase area. In this area, the material is single-phase, without NTE.

The experimental curves 3 (decay of the ***t***−*ss*) and 4 (decay of the ***β***−*ss*) [2] in the subsolidus region of the GdF_3_-TbF_3_ system (black dots and red circles in Figure 2) were approximated using second-degree polynomials (6) and (7):

***T*** = −169.6 *x*^2^ + 322.2 *x* + 1069.7 (6)

***T*** = 88.4 *x*^2^ + 182.6 *x* + 1066.5 (7)

Based on the described methodology, using Equations (6) and (7), it is possible to calculate the volume–temperature dependencies for any of the 34 *R*F_3_-*R′*F systems studied experimentally [2].

Temperature is an active factor controlling ΔV/V, which is one of the parameters of NTE-II material prospects. The second factor is passive, that is, a fixed composition, *x*. In our case, this is the composition *x*_o_. If ΔV/V is approximately estimated via the scope of a material application, the composition can be changed in two ways. Small changes within the two-phase area are achievable in one system. For large changes, another system is searched using the *R*F_3_-*R′*F_3_ array [16].

### 2.3. NTE-II in the GdF_3_-TbF_3_ System

The construction of a volume–temperature dependence is widely used in various fields of research to calculate the NTE for various purposes. ΔV/V = ***f***(***T***) dependence was used [1] to determine the CTE. We present the calculation of such dependencies for Gd_1−x_Tb_x_F_3_ at several *x*.

#### 2.3.1. The ΔV/V = ***f***(***T***) Dependencies at the PolTr in Gd_1−x_Tb_x_F_3_

**Figure 3:**
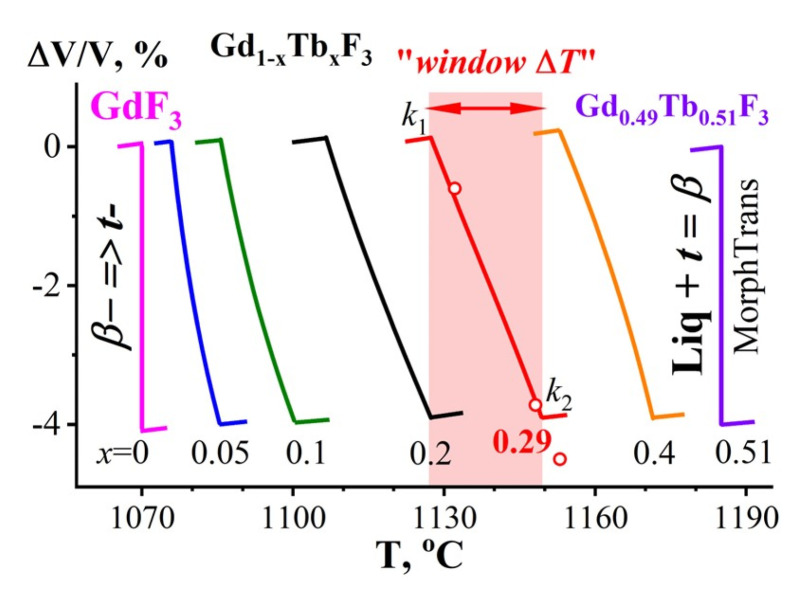
The ΔV/V = ***f***(***T***) dependencies for Gd_1−x_Tb_x_F_3_ (*x*_o_ = 0 (magenta line), 0.05 (blue line), 0.1 (green line), 0.2 (black line), 0.29 (red line), 0.4 (orange line), 0.51 (lilac line)).

The ΔV/V = ***f***(***T***) dependencies at the ***β***− → ***t***− PolTr in Gd_1−x_Tb_x_F_3_ for *x*_o_ = 0, 0.05, 0.1, 0.2, 0.29, 0.4 and 0.51 are shown in Figure 3.

Equation (8) is used to construct the curves:

ΔV/V = (V_(***t***+***β***)_ − V***_t_***_−_)/V***_t_***_−_ (8)

where V_(***t***+***β***)_ is the two-phase composite volume at ***T***′ and V***_t_***_−_ is the V_form_ of the ***t***− type phase at the end point of the PolTr at ***T_t_***_−ss_ (insert in Figure 2).

ΔV/V for (***β*** + ***t***)-Gd_1−x_Tb_x_F_3_ changes within Δ***T***_trans_ = ***T_t_***_−ss_-***T_β_***_−ss_ (the “window Δ***T***”) from 0 to 4.22%. The (***β*** + ***t***)-Gd_1−x_Tb_x_F_3_ composites have NTE-II over the “window Δ***T***”. ΔV/V is decreasing with increasing ***T***, and the CTE is negative.

Beyond the “window Δ***T***”, (***β*** + ***t***)-Gd_1−x_Tb_x_F_3_ refers to conventional materials without NTE and with the positive CTE.

The TbF_3_ contents at the starting points of the ***β***-Gd_1−x_Tb_x_F_3_ → ***t***-Gd_1−x_Tb_x_F_3_ PolTr at ***T_β_***_−ss_ are indicated by *x*_o_ = 0, 0.05, 0.1, 0.2, 0.29, 0.4, 0.51 numbers under the curves. The dependencies are constructed within the Δ***T***_trans_ = ***T_t_***_−**ss**_-***T_β_***_−**ss**_ (the “window Δ***T***”), which is highlighted in light red in the diagram in Figure 3 for *x* = 0.29.

##### The PolTr in GdF_3_ and Gd_0.49_Tb_0.51_F_3_ and NTE-II

The vapor pressure (***P***) for all *R*F_3_, except ScF_3_, is low. This allows us to neglect ***P*** and consider all *R*F_3_ discussed here to be condensed systems with invariant PolTrs with ***T***_trans_ = *const.*

For single-component GdF_3_, there is the invariant PolTr of the first kind at point 1 (Figure 2). At ***T***_trans_ = *const*, the two solid phases are in equilibrium ***β***-GdF_3_ ↔ ***t***-GdF_3_. The PolTr is accompanied by a giant volume contraction (ΔV/V ~ −4.3% [14,15,19]). The GdF_3_ component has no measurable CTE at PolTr.

The ΔV/V = ***f***(***T***) curve for GdF_3_ (*x* = 0) in Figure 3 shows the absence (within the measurement error) of a slope between the ***β***-GdF_3_ and ***t***-GdF_3_ components. The shape of the curve for GdF_3_ fully corresponds to the first scheme from [1] (Figure 1a) and to the phase rule.

The curve for the two-component Gd_0.49_Tb_0.51_F_3_ *ss* (*x* = 0.51 in Figure 3) also has no slope (Δ***T***_trans_ = 0) because the PolTr of this composition represents an invariant process, a peritectic phase reaction.

##### The PolTr in Two-Phase (***β*** + ***t***)-Gd_1−x_Tb_x_F_3_ Composites and NTE-II Materials

The ΔV/V = ***f***(***T***) curves for the two-phase (***β*** + ***t***)-Gd_1−x_Tb_x_F_3_ composites with 0.05 < *x* < 0.40, calculated using (2)–(8), have a slope (Figure 3), since their PolTrs represent monovariant processes and occur in the interval of Δ***T***_trans_ > 0.

The ΔV/V change within the two-phase area of the phase diagram (insert in Figure 2) consists of: (1) the changes in V***_β_***_−*ss*_ and V***_t_***_−*ss*_ with the changes in *ss* compositions along curves 3 and 4, (2) the differences in V***_β_***_−*ss*_ and V***_t_***_−*ss*_ of the two structural types (***β***−*ss* and ***t***−*ss*) at ***T***′ of the selected conode (*k*_3_ in the insert, Figure 2), and (3) the quantitative ratio of the ***β***−*ss* and ***t***−*ss* phases (according to the lever rule) in the NTE-II composite on isothermal sections with ***T***′ = *const* (the *k*_3_ conode).

This is the thermodynamic mechanism for controlling the parameters of materials via isomorphism in any “temperature—composition” (***T*** − *x*) system. It follows from the analysis of the equilibrium phase diagram of a binary system and is not related to the sign of the ΔV/V change at the PolTr and NTE-II.

Owing to its fundamental thermodynamic nature, the mechanism of formation of two-phase composites is universal for compounds of any chemical class of substances. The volume reduction at the PolTr (when heated) makes the composite an NTE-II material.

##### The PolTr in the Two-Phase Composite Gd_0.71_Tb_0.29_F_3_ (Gross Composition) According to the Phase Diagram and the NTE-II Parameters

To describe an NTE-II material with the “window Δ***T***” formed in the GdF_3_-TbF_3_ system, the gross composition of Gd_0.71_Tb_0.29_F_3_ was selected. It is obtained by averaging the compositions of the three experimental points in the phase diagram (indicated by red circles in Figure 2). It corresponds to the dashed vertical ***I*** in Figure 2.

On the curve *x* = 0.29 in Figure 3, three V_form_ values (red circles) obtained from the experimental points of the phase diagram (also red circles in Figure 2) are plotted. The concentration *x* = 0.29 is the average for these points.

The ΔV/V values obtained from the experimental data (red circles) of the phase diagram correlate well with the calculated ΔV/V = ***f***(***T***) dependencies described by lines 3 and 4, between *k*_1_ and *k*_2_.

#### 2.3.2. The ΔV/V = *f*(Δ***T***) Dependence for the (***β*** + ***t***)-Gd_1−x_Tb_x_F_3_ Composite

**Figure 4:**
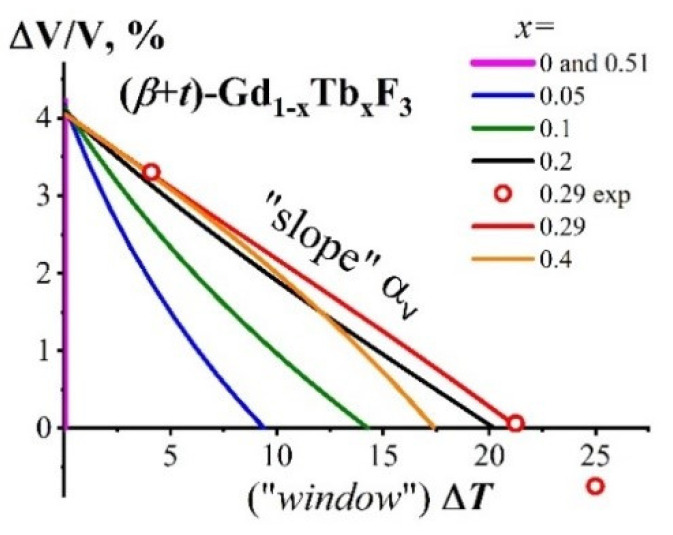
The ΔV/V = ***f***(Δ***T***) dependencies for (***β*** + ***t***)-Gd_1−x_Tb_x_F_3_ (*x* = 0, 0.05, 0.1, 0.2, 0.29, 0.4, and 0.51). The points calculated from the experimental data are indicated by opened red circles.

To characterize the CTE of (***β*** + ***t***)-Gd_1−x_Tb_x_F_3_ two-phase composites formed in the GdF_3_-TbF_3_ system, the dependencies of the relative volume change ΔV/V on Δ***T*** were constructed. The ΔV/V = ***f***(Δ***T***) dependencies for (***β*** + ***t***)-Gd_1−x_Tb_x_F_3_ (*x* = 0, 0.05, 0.1, 0.2. 0.29, 0.4, 0.51) are shown in Figure 4.

The ΔV/V values for the curves in Figure 4 were calculated using (2)–(8). The temperature interval Δ***T*** is calculated using (9) for every ***T***’ within the temperature interval of the ***β***-Gd_1−x_Tb*_x_*F_3_ → ***t***-Gd_1−x_Tb*_x_*F_3_ PolTr (when heated).

Δ***T*** = ***T***’ − ***T_β_***_−*ss*_ (9)

The three ΔV/V values obtained from the experimental data with *x* = 0.29 (opened red circles) are shown in Figure 4.

For *x* = 0 and 0.51 with invariant equilibrium, the dependencies in Figure 4 represent vertical lines (the “window Δ***T***” = 0). They coincide with each other and are marked in purple.

For the two-phase composite (***β*** + ***t***)-Gd_1−x_Tb_x_F_3_ (0 < *x* < 0.51), the ΔV/V = ***f***(Δ***T***) dependencies are nonlinear and have different forms for different *x* values. The CTE of (***β*** + ***t***)-Gd_1−x_Tb_x_F_3_ varies within the “window Δ***T***”.

#### 2.3.3. The Dependence of Δ***T***_trans_ (the “Window ΔT”) on a Chemical Composition for (***β*** + ***t***)-Gd_1−x_Tb_x_F_3_ Composites

**Figure 5:**
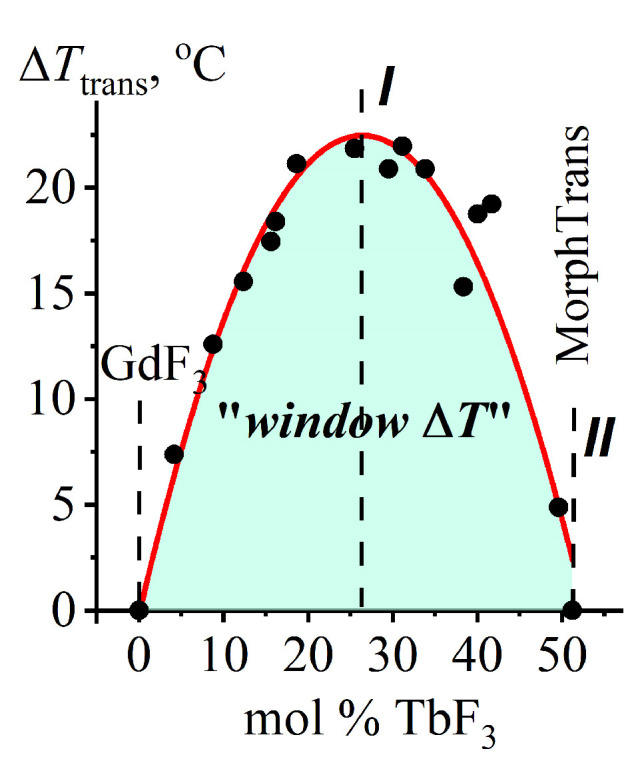
The Δ***T***_trans_ = ***f***(*x*) dependence for (***β*** + ***t***)-Gd_1−x_Tb_x_F_3_ (*x* = 0, 0.05, 0.1, 0.2, 0.29, 0.4, and 0.51). The calculated data are shown as black circles and the approximated curve is represented by a red line. The area of the ***β***−*ss* → ***t***−*ss* PolTr in Gd_1−x_Tb_x_F_3_ (0 < *x* < 0.51) is shown in light green.

The dependence of Δ***T***_trans_ (the “window Δ***T***”) on the (***β*** + ***t***)-Gd_1−x_Tb_x_F_3_ composition *x* is shown in Figure 5. It is calculated for the area between curves 3 and 4 (Figure 2). The area of the ***β***−*ss* → ***t***−*ss* PolTr in Gd_1−x_Tb_x_F_3_ (0 < *x* < 0.51) is shown in light green in Figure 2 and Figure 5.

The curve was approximated using a second-degree polynomial (10):

Δ***T***_trans_ = −325.3 *x*^2^ + 171.6 *x* − 0.184 (10)

The approximated curve is shown in Figure 5 with a red line. It has a maximum of Δ***T***_trans_ = 22 °C at *x* = 0.26.

The value of Δ***T*** (the “window Δ***T***“) in the GdF_3_-TbF_3_ system corresponds to the maximum (Δ***T*** = 22 °C) on the curve in Figure 5. This is much higher than the reproducibility of the ***T*** measurements in *R*F_3_-*R′*F_3_ systems (±3 °C).

## 3. Materials and Methods

### Two-Component NTE-II Materials in RF_3_-R′F_3_ and MF_2_-RF_3_ Systems (M = Ca, Sr, Ba; R = La − Lu)

The necessary and sufficient features (polymorphism with the density anomaly) for two-component composite NTE-II materials in the GdF_3_-TbF_3_ system can be applied to any *R*F_3_-*R′*F_3_ system.

The two-phase (***t***−*ss* + ***β***−*ss*) composites are NTE-II materials with adjustable parameters. The potential for using the material is estimated using the parameter of the average volume change ΔV/V_av_. The V_av_ at a fixed gross composition of a system is determined by the ***β***−*ss* and ***t***−*ss* decay (synthesis) curves and the temperature ***T***. The regulation of ΔV/V_av_ is achieved by changing ***T*** within the “window Δ***T***”. The available Δ***T*** values are determined using phase diagrams.

There are 50 such systems [16]. Phase diagrams of 11 types of systems with the giant NTE-II were studied: GdF_3_-TbF_3_, GdF_3_-DyF_3_, GdF_3_-HoF_3_, LaF_3_-GdF_3_, CeF_3_-GdF_3_, PrF_3_-GdF_3_, NdF_3_-GdF_3_, GdF_3_-ErF_3_, GdF_3_-TmF_3_, GdF_3_-YbF_3_, and GdF_3_-LuF_3_ [2].

Berthollide phases with the ***t***− type structure formed in *M*F_2_-*R*F_3_ systems with *M* = Ca, Sr, Ba; *R* = Gd – Lu, Y, and NaF-*R*F_3_ with *R* = Gd, Tb [2] are also composite materials with NTE-II. The temperature interval “window Δ***T***” of the two-phase region (***t***−*ss* + ***β***−*ss*) of berthollide phases is more than 300 K.

The “window Δ***T***” achievable in *R*F_3_-*R′*F_3_ systems is comparable with the “window Δ***T***” of conventional NTE materials. The “window Δ***T***” parameter plays a decisive role in controlling the performance characteristics of NTE-II materials when they are used as thermal expansion compensators in high tech. To date, no fluoride materials (single- or multicomponent) have been tested for use in this field.

The NTE-II materials *R*F_3_, *R*_1−x_*R*′_x_F_3_ and berthollide phases based on *R*F_3_ are the best superionic conductors with fluorine-ion conductivity [21,22,23,24,25,26,27,28]. They are also used as scintillators [29,30,31] and lasers [32,33] and have a low refractive index dispersion [34].

## 4. Conclusions

The thermodynamic mechanism for the formation of two-component, two-phase NTE-II materials with controllable properties in a binary system is described. It follows from the equilibrium phase diagram. Owing to its fundamental nature, this mechanism is universal for compounds of any chemical class of substances. 

A necessary and sufficient condition for the formation of a material with NTE-II is a combination of polymorphism and density anomaly. Under comparable thermal conditions, the V_high_ of the high-temperature form is lower than that of the low-temperature form V_low_.

The “operating temperature window Δ***T***” determines the range of compositions and temperatures of the existence of the two-phase composite NTE-II material in a binary system. Its dimensions are given by the monovariant decay (formation) curves of *ss*, with the structures giving the density anomaly at the PolTr.

The volume change at the PolTr characterizes the NTE-II potential of a material in a particular system.

Polymorphism in *R*F_3_ (*R* = Pm, Sm, Eu, and Gd) occurs between the main structural types of *R*F_3_: β-YF_3_ (***β***-low-temperature form) and ***t***-LaF_3_ (***t***-high-temperature form).

*R*F_3_ (*R* = Pm, Sm, Eu, and Gd) represents a new class of fluoride single-component NTE-II materials. They possess the giant NTE of the 2nd type within the “window Δ***T***” = 0 temperature range.

Isomorphism in *R*F_3_-*R′*F_3_ systems chemically modifies *R*F_3_ to form two-component *R*_1−x_*R′*_x_F_3_ materials. *R*_1−x_*R′*_x_F_3_ have the giant NTE-II if one or both of their components belong to the “mother” single-component dimorphic *R*F_3_ with *R* = Pm, Sm, Eu, and Gd.

*R*_1−x_*R′*_x_F_3_ with *R* = Pm, Sm, Eu, and Gd expands a new class of fluoride two-component NTE-II materials. They possess the giant NTE of the 2nd type within the “window Δ***T***” > 0 temperature range in which the two-phase composites (***β*** *+****t***)-*R*_1−x_*R′*_x_F_3_ are formed between the two monovariant decay (formation) curves of *R*_1−x_*R′*_x_F_3_ *ss*. Beyond the “window Δ***T***” *R*_1−x_*R′*_x_F_3_ are conventional materials without NTE.

For the GdF_3_-TbF_3_ model system, the schemes describing the PolTr in *R*F_3_-*R*′F_3_ systems are presented. The equations for calculating the formula volumes of the ***β***-Gd_1−x_Tb_x_F_3_-*ss* (V***_β_***_−_) and ***t***-Gd_1−x_Tb_x_F_3_-*ss* (V***_t_***_−_) and their percentages in the (***β*** + ***t***)-Gd_1−x_Tb_x_F_3_ two-phase composite at the PolTr are obtained.

For the GdF_3_-TbF_3_ model system, according to the experimental data, the ΔV/V = ***f***(***T***), ΔV/V = ***f***(Δ***T***) and the “window Δ***T***” = ***f***(*x*) dependencies are calculated.

The scheme for calculating the specific parameters of NTE-II materials presented in this work can be applied to any *R*F_3_-*R′*F_3_ system.

The “window Δ***T***” achievable in *R*F_3_-*R′*F_3_ systems is comparable with the “window Δ***T***” of conventional NTE materials. This parameter plays a decisive role in controlling the performance characteristics of NTE-II materials when they are used as thermal expansion compensators in high-tech applications.

## Data Availability

The data presented in this study are available on request from the corresponding author.
